# A novel *SLC20A2* mutation presenting with paroxysmal kinesigenic dyskinesia and epilepsy in a Chinese patient: a case report

**DOI:** 10.1007/s13760-023-02182-5

**Published:** 2023-01-26

**Authors:** Lijun Wang, Jianfang Ma, Xiangqian Che

**Affiliations:** 1 Department of Neurology and Institute of Neurology, Ruijin Hospital, Shanghai Jiao Tong University School of Medicine, Shanghai, 200025 China; 2 Department of Neurovascular Center, Changhai Hospital, Naval Medical University, Shanghai, 200433 China

Dear Sir,

*SLC20A2* is the most common and the main causative gene of primary familial brain calcification (PFBC), which is a rare degenerative neurological disorder characterized by bilateral symmetrical calcifications in the basal ganglia, thalamus, subcortical white matter, cerebellum, and other brain regions. The patients possess a variety of manifestations including movement disorders, cognitive impairment, and psychiatric symptoms. Here, our study demonstrated a novel pathogenic heterozygous *SLC20A2* mutation causing PFBC and manifesting as both paroxysmal dyskinesia and epilepsy in one patient, which expanded and enriched the phenotypic spectrum of a single *SLC20A2* variant.

## Case presentation

**Fig. 1:**
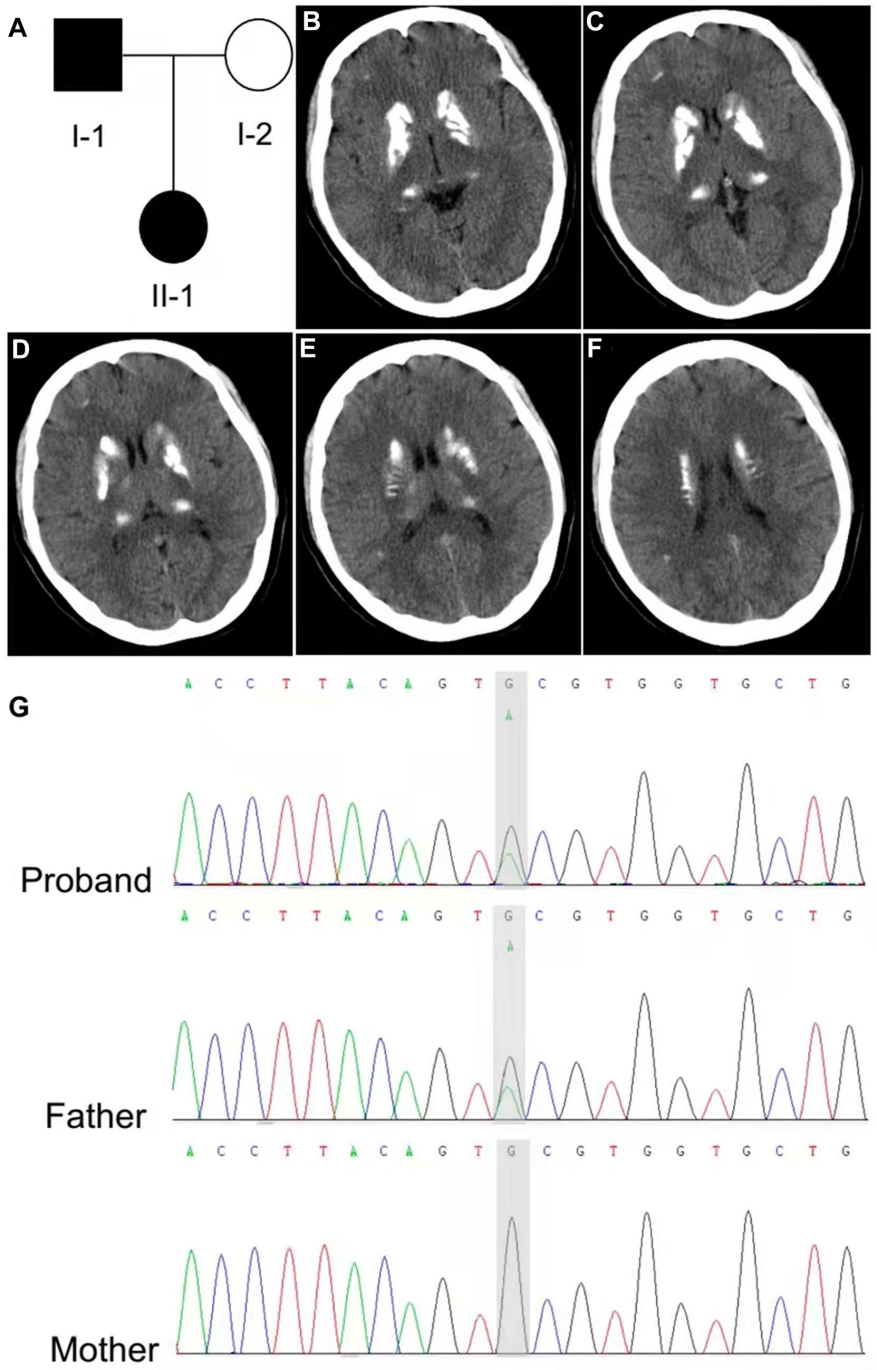
CT scans and genetic findings. **A** Pedigree of the patient’s family with *SLC20A2* mutation. The black filled-in symbols represent the affected individuals. Empty symbols indicate unaffected individuals. The proband is II-1. **B**–**F** Cranial neuroimaging. The brain CT showed multiple calcifications in the basal ganglia, thalamus and frontal parietal lobe on both sides. **G** DNA sequence revealed a novel site mutation of *SLC20A2* (NM_001257180.2: exon10, c.1786 C > T, p. His 596Tyr)

A 22-year-old female patient (Fig. 1A, II-1) revealed an eight-year history of intermittent paroxysmal episodic involuntary movement and was admitted to our hospital for generalized tonic–clonic seizures (GTCS) resulting in a loss of consciousness lasting 10 min without any obvious inducement for the first time. The involuntary movement attacks were induced by sudden voluntary actions, such as sudden standing, mainly in the right upper limb, and occurred up to 10 times per day. Her general physical and neurological examinations were normal. The patient was 158 cm in height, weighed 53 kg. There was no history of neurological disease, brain tumor, or trauma. One month ago, the patient terminated the pregnancy due to the discovery of fetal polycystic kidney disease on routine malformation screening. After signing written informed consents, a series of auxiliary examinations were arranged for the family. Laboratory tests showed normal levels of serum calcium, serum phosphorus, thyroid function, liver function, kidney function, blood glucose, blood clotting function, serum creatine phosphokinase, rheumatic immune-related antibodies, folate. Parathyroid hormone-related peptide was 90.3↑ pg/mL, 25-OH-VitD was 15.51↓ nmol/L. Brain CT scan showed multiple calcifications in the basal ganglia, thalamus, and frontal parietal lobe on both sides (Fig. 1B–F). Color ultrasound of thyroid, parathyroid, and cervical lymph nodes showed no obvious abnormalities. The electroencephalogram (EEG) indicated, isolated sharp waves in the left prefrontal temporal lobe. Levetiracetam was increased to 0.75 g twice daily due to poor control of epilepsy. However, treatment with 12.5 mg of lamotrigine, there was no recurrence of abnormal movements or seizures during one-year follow-up period.

**Fig. 2:**
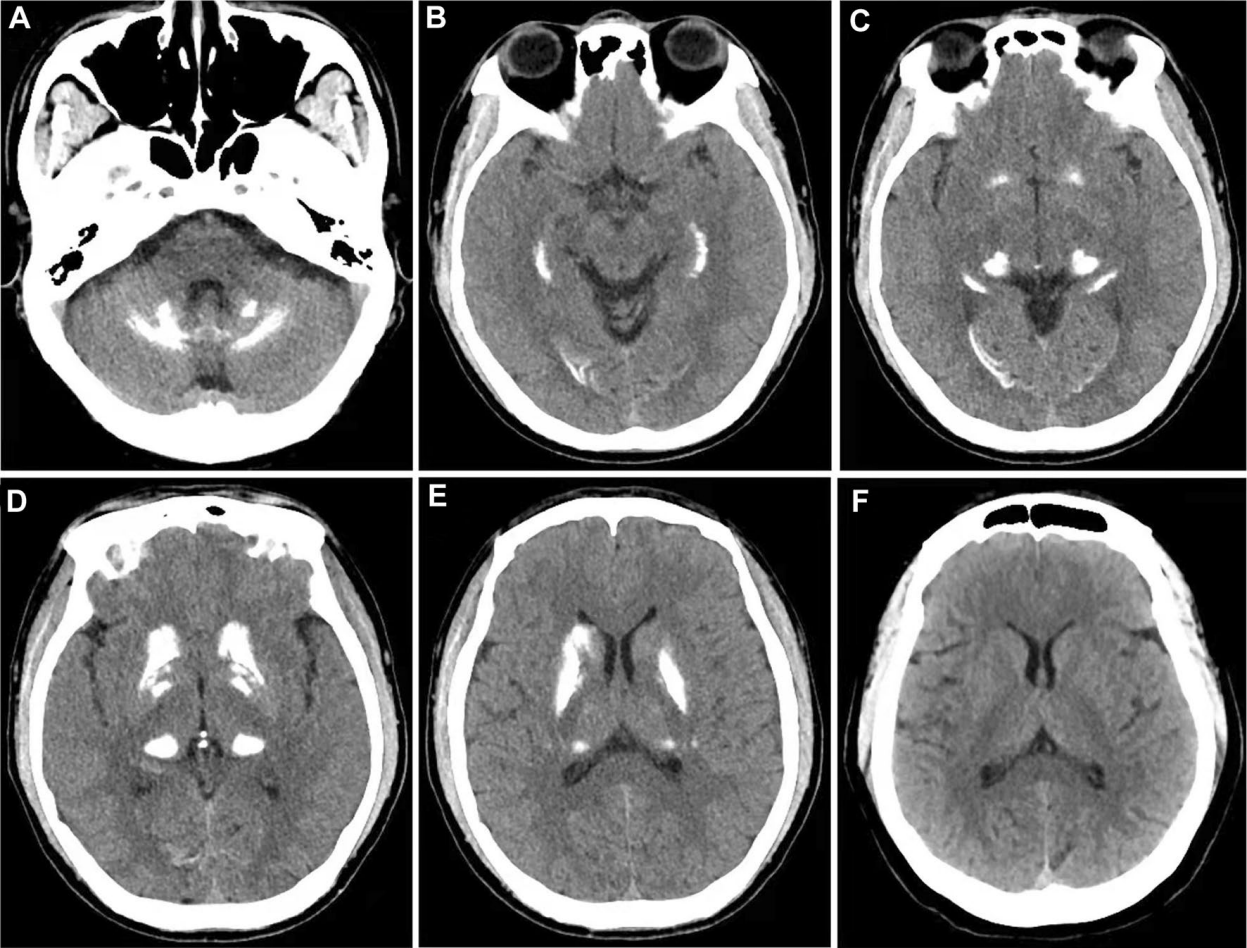
Cranial neuroimaging. **A**–**E** Cranial CT of the proband’s father showed multiple scattered high-density shadows in bilateral basal ganglia, thalamus, cerebellar and subcortex. **F** Cranial CT of the proband’s mother is normal

The brain CT of the proband’s father showed multiple scattered high-density shadows in bilateral basal ganglia, thalamus, cerebellar and subcortex, which might be metabolic diseases (Fig. 2A–E), but he remained asymptomatic. While the brain CT of her mother was normal (Fig. 2F). Finally, we identified a novel heterozygous variant (NM_001257180.2: exon 10, c.1786 C > T, p. His 596Tyr) of *SLC20A2* through whole-exome sequencing of the proband. Further Sanger sequencing verification revealed that this mutation was inherited from her father, and the proband and her father are both heterozygous carriers (Fig. 1G).

## Discussion

*SLC20A2* encoding type III sodium-dependent phosphate transporter 2 (PiT2), was identified as the most common, which may be the main causative gene of PFBC in China. Here, we firstly reported a novel *SLC20A2* mutation with both paroxysmal kinesigenic dyskinesia (PKD) and epilepsy in one patient as the main symptoms. Wang et al. [1] identified a *SLC20A2* heterozygous c.1784C > T (p. Thr595Met) variant in familial idiopathic basal ganglia calcification with in vitro functional support. Our study identified c.1786 C > T (p. His596Tyr) variant of *SLC20A2*, which is in the same highly conserved residue (1 amino acid apart). The mutation is also not found in any public database, predicted to be damaging in silico analysis; therefore, this mutation is highly likely to be pathogenic. Our *SLC20A2*-related PFBC case expanded the phenotypic spectrum, which reinforced the associations of the *SLC20A2* gene with the etiopathology of PFBC, and suggested a potential pathophysiological connection between PKD and epilepsy.

Due to highly phenotypic heterogeneity, the *SLC20A2* mutation patients possess a variety of manifestations including movement disorders, cognitive impairment, and psychiatric symptoms. But epilepsy was rarely reported. Knowles et al. [2] described a pediatric patient with PFBC and refractory epilepsy, accompanied by generalized interictal discharge and focal seizure. Knowles’s patient had a pathogenic *SLC20A2* mutation, and a heterozygous *SCN2A* variant of unknown significance, which he inherited from his father who remained asymptomatic. The brain’s ability to restore calcification is considered to be the most probable cause for PFBC patients to remain asymptomatic. Fjaer et al. [3] described two siblings with PFBC due to a variant in *SLC20A2* and only generalized tonic–clonic seizures as the phenotypic characteristics. The rate of epilepsy in PFBC (5–10%) is reported to be higher than that of the general population (0.5–1%), indicating that PFBC is a risk factor for epilepsy.

Until now, 12 PFBC cases in 6 unrelated families manifested as PKD have been reported [4]. In a previous study in Chinese population, Zhu et al. [5] described a familial idiopathic basal ganglia calcification caused by the *SLC20A2* gene mutation (c.1086delC) who manifest as juvenile onset PKD. The observation is similar to our case: a patient has basal ganglia calcification but is asymptomatic, with other patients manifesting PKD at the age of 11–14 years. PKD is characterized by recurrent and transient episodes of involuntary movements precipitated by a sudden voluntary action. The age at onset generally ranges from 7 to 15 years, while our patient at 14 years. Unlike epilepsy, the PKD attacks have a clear kinesigenic trigger and the individuals remain conscious during the attack, which can be used to distinguish between the two disorders. The calcification in the basal ganglia may have an impact on the sensory processing, or the basal ganglia-thalamo-sensorimotor cortical circuitry, which finally cause the involuntary movement. Therefore, the calcification of the basal ganglia that cause the manifestation of PKD has the anatomical basis.

There is a dramatic response to low doses of lamotrigine in our case, for the reason that lamotrigine could inhibit Na^+^ channel activation in various cell systems. Although the patient’s father had calcifications in neuroimaging, he was not affected by PKD or epilepsy. Despite substantial brain calcifications, individuals may remain asymptomatic clinically or they could develop symptoms afterwards. Further studies are needed to provide deeper insights into the pathomechanism underlying the clinic-genetic heterogeneity of PFBC.

## Conclusion

In conclusion, the present case reported a novel pathogenic heterozygous *SLC20A2* mutation causing PFBC and manifesting as both PKD and epilepsy in a Chinese patient. This case demonstrates the clinical heterogeneity of PFBC and enriched the phenotypic spectrum of *SLC20A2*.

## Data Availability

All datasets generated for this study are included in the article. The figures in our paper are original for this article, and we have permission to use it.
